# Balloon Aortic Valvuloplasty in Patients Admitted for Cardiogenic Shock with Severe Aortic Stenosis: A Retrospective Analysis of 14 Cases

**DOI:** 10.7759/cureus.5407

**Published:** 2019-08-17

**Authors:** Miguel L Varela, Pedro Teixeira, Marta Ponte, Daniel Caeiro, Adelaide Dias, Alberto Rodrigues, Pedro Braga

**Affiliations:** 1 Critical Care Medicine, Intensive Care Medicine 1, Hospital De Faro, Faro, PRT; 2 Cardiology, Centro Hospitalar Vila Nova De Gaia, Gaia, PRT

**Keywords:** balloon aortic valvuloplasty, cardiogenic shock, severe aortic stenosis, tavi, cardiac critical care

## Abstract

Introduction

Balloon aortic valvuloplasty (BAV), introduced in 1986, quickly lost its wide adoption due to the high incidence of restenosis after the procedure and due to improved skills in transcatheter aortic valve implantation (TAVI). It has seen a re-emergence in the last few years as bailout therapy in critical care patients presenting with cardiogenic shock (CS) and severe aortic stenosis (AS), who are temporarily unable to tolerate such a procedure as TAVI or surgery for valve replacement.

Methods

We did a retrospective analysis of every BAV performed between January 1, 2008, and November 11, 2018, in our hospital and identified those admitted to the cardiac intensive care unit (CICU) due to cardiogenic shock with severe aortic stenosis, as defined in the European Society of Cardiology Guidelines. Procedures were categorized as emergent (within 24h after the decision to intervene) and urgent (24h after the decision was made but before discharge).

Results

During this period, of 98 BAV performed, 14 were performed on patients with CS with severe AS, nine of them being emergent. The patients’ mean age was 76.2±7.2 years, with 6 (43%) of them being female.

On the day of BAV, the mean Euroscore II and sequential organ failure (SOFA) were, respectively, 19±7% and 8.0±2.4 in emergent cases and 11±5% and 4.8±2.9 in urgent cases. In patients deemed emergent, there was a tendency for a decrease in SOFA in the days following the procedure, although not statistically significant (p>0.05). Clinically significant aortic regurgitation did not occur in any patient, neither were there any major post-procedure complications. Thirty-day mortality was 33% in emergent cases and 0% in urgent cases.

In emergent cases, four were later submitted to TAVI and one had surgery for aortic valve replacement surgery. Only one patient in the urgent group was regarded as a candidate for TAVI.

Discussion and conclusion

Emergent cases presented with higher scores of severity and procedure risk and had greater mortality. In this group, a greater proportion of survivors was later deemed fit for definite procedures. This highlights that presenting status does not seem to influence the prognosis of those extremely high-risk patients once the acute event is promptly treated. Nevertheless, the low sample size precludes generalization of the findings.

BAV as bailout treatment may be safe in patients presenting with CS and severe AS, allowing patient survival for elective definitive treatment.

## Introduction

Cardiogenic shock is a cardiac disorder that results in clinical and biochemical evidence of tissue hypoperfusion [1]. Aortic stenosis (AS) is one of the many causes of cardiogenic shock, as it causes obstruction to left ventricular outflow [2], complicated by an already hypertrophied left ventricle, due to chronically increased afterload, with resulting systolic and diastolic dysfunction and reduced coronary flow reserve (“burn out” phase) [3-4]. Attempts to treat medically patients presenting with cardiogenic shock and severe aortic stenosis are largely unsuccessful, with high mortality [3]. Yet, these patients also present a high surgical risk for aortic valve replacement (AVR) [5].

Balloon aortic valvuloplasty (BAV) was first described by Cribier in 1986 as an alternative to valve replacement in AS [6]. Later, in 1992, the same author described its emergent use for patients with AS presenting with cardiogenic shock, resulting in significant improvement in the cardiac index and aortic transvalvular gradients [7]. Despite this early enthusiasm, mid- and long-term outcomes proved unsatisfactory: restenosis rate was high (70%), peri-procedural complications were noteworthy, and one-year mortality was no different from conservative treatment [8-11]. Additionally, with the introduction of transcatheter aortic valve implantation (TAVI) in 2002, which conferred long-lasting results comparable to those of surgical replacement of the aortic valve, in low to high surgical risk individuals, BAV feels out of use [12-14]. However, TAVI requires a larger sheath size than that for BAV, takes longer, and requires accurate imaging for successful implantation, features that may preclude its use in emergent settings [15-16]. Its associated costs should also be considered. Hence, BAV was recently revived as a procedure to be considered in hemodynamically unstable patients as a bridge to definite treatment, as recommended in the European Society of Cardiology’s 2012 guidelines [17]. Recent technical improvements, such as smaller sheath sizes and newer balloon formats, significantly reduced procedural complications [18-19]. The numbers clearly show a yearly increase in BAVs being performed [20]. A BAV may also be used for palliative purposes [15].

We aimed to review the experience of our hospital with patients with cardiogenic shock and severe AS treated with BAV, which is one of the main referral hospitals in Portugal for structural cardiac interventions.

## Materials and methods

Data gathering

A search was performed on our hospital’s database on cardiology procedures for all BAV procedures, and then each patients’ electronic file was analyzed. For those patients referenced from other hospitals for procedures in our hospital, data was obtained from available information in our national inter-hospital data sharing registry.

Patient selection

We collected all the BAV procedures performed at our center between January 1, 2008, and December 31, 2018. Of those, we identified those patients admitted to the cardiac intensive care unit (CICU) due to cardiogenic shock with severe AS that performed BAV before or after being admitted. Procedures were categorized as emergent (within 24h after the decision to intervene) or urgent (24h after the decision was made but before discharge).

Cardiogenic shock was diagnosed if the following three criteria were fulfilled: systolic blood pressure <90 mmHg for >30 min or vasopressors required to achieve a blood pressure ≥90 mmHg; pulmonary congestion or elevated left-ventricular filling pressures; signs of impaired organ perfusion with at least one of the following: (a) altered mental status; (b) cold, clammy skin; (c) oliguria; (d) increased serum-lactate [1]. Severe AS was defined according to the European Society of Cardiology guidelines [21].

Technical characteristics of BAV procedures

BAV procedures were guided by fluoroscopy; using rapid pacing, an aortic balloon introduced through the femoral artery. The device used for dilation was the Loma Vista Medical TRUE Dilatation™ balloon valvuloplasty catheter (California, US). Vascular closure was made using mechanical compression and/or a Proglide device (Abbott Laboratories, Chicago, US). Transaortic pressures were recorded before and after valvuloplasty in the cardiac catheterization laboratory. The reader can consult the review by Keeble et al. for further explanation on the procedure [10].

Statistical analysis

Continuous variables were displayed as means with standard deviation. Results for categorical variables were expressed as frequencies and percentages. We checked for the normality of each distribution using the Shapiro-Wilk test and histograms. If the normality of the distributions was confirmed, the t-student two-sample test was used, if not, the Mann-Whitney U test was used. Statistical significance was obtained when the p-value was below 0.05.

For data analysis, we used R Commander version 2.5.1 in the 3.5.1 (64-bit) version of the R environment and SPSS version 25 (IBM Corporation, Armonk, NY, US).

## Results

Of all 98 BAV performed during the study period, 14 were performed on patients with cardiogenic shock and severe AS. Nine procedures were classified as emergent, and five as urgent. Results are summarized in Table 1 and Table 2.

**Table 1:** Patient’s characteristics, urgent cases (n=5) + – mild; ++ – moderate; +++ – severe; AF – atrial fibrillation; AMI – acute myocardial infarction; AS – aortic stenosis; AR – aortic regurgitation; AVR – aortic valve replacement; BAV – balloon aortic valvuloplasty; CICU – cardiac intensive care unit; CKD – chronic kidney disease; COPD – chronic obstructive pulmonary disease; DM – diabetes mellitus; DP – dopamine; EF - ejection fraction; HF – heart failure; IMV – invasive mechanical ventilation; MTG - mean transaortic gradient; MR – mitral regurgitation; NA – noradrenaline; NYHA - New York Heart Association; PTG- peak transaortic gradient; TAVI – transcatheter aortic valve implantation; TR – tricuspid regurgitation; U – urgent; * value obtained from the echocardiogram

Patient #	Age	Sex	Main comorbidities		Baseline EF	Emergent / urgent	Number of days			Vasoactive agents in the day of BAV	IMV in the day of BAV	30-day status	1-year status	2-year status	Cause of death	TAVI / AVR (# days after BAV)	PTG before / after	MTG before / after
			General	Valve disease (other than AS)			From hospital admittance to CICU admittance	In the CICU	After discharge from the CICU									
U1	69	M	Hypertension, Type 2 DM, HF NYHA 4	TR +++	Severe depression	Urgent	20	13	0	-	-	Alive	Dead	-	?	TAVI (7)	43 / 9	35 / 10
U2	68	M	Hypertension, Asthma, HF, COPD	TR + MR ++	Severe depression	Urgent	11	6	174	NA	-	Alive	Dead	-	Pneumonia	-	68 / 30	52 / 33
U3	82	F	Hypertension, Type 2 DM, HF, AF, COPD	TR +++ MR +	Severe depression	Urgent	0	5	13	-	-	Alive	Dead	-	?	-	35 /23	24 / 24
U4	72	F	Hypertension, Type 2 DM, AF, HF	TR ++ MR ++	?	Urgent	3	4	6	-	-	Alive	Dead	-	?	-	30 / 13	16 / 13
U5	82	F	Hypertension, Type 2 DM, AF, HF	TR ++ MR ++	Preserved	Urgent	13	1	18	-	Yes	Alive	Alive	?	-	-	39 / 18	? / 7

**Table 2:** Patient’s characteristics, emergent cases (n=9) + – mild; ++ – moderate; +++ – severe; AF – atrial fibrillation; AMI – acute myocardial infarction; AS – aortic stenosis; AR – aortic regurgitation; AVR – aortic valve replacement; BAV – balloon aortic valvuloplasty; CICU – cardiac intensive care unit; CKD – chronic kidney disease; COPD – chronic obstructive pulmonary disease; DM – diabetes mellitus; DP – dopamine; E – emergent; EF - ejection fraction; HF – heart failure; IMV – invasive mechanical ventilation; MTG - mean transaortic gradient; MR – mitral regurgitation; NA – noradrenaline; PTG- peak transaortic gradient; TAVI – transcatheter aortic valve implantation; TR – tricuspid regurgitation; * value obtained from the echocardiogram

Patient #	Age	Sex	Main comorbidities		Baseline EF	Emergent / urgent	Number of days			Vasoactive agents in the day of BAV	IMV in the day of BAV	30-day status	1-year status	2-year status	Cause of death	TAVI / AVR (# days after BAV)	PTG before / after	MTG before / after
							From hospital admittance to CICU admittance	In the CICU	After discharge from the CICU									
E1	68	F	Hypertension, CKD stage IV, HF	TR ++ MR ++ AR +	?	Emergent	1	9	8	-	-	Alive	Alive	Alive	-	TAVI (6)	79* / 35	49* / 27
E2	79	F	-	MR +++	Moderate depression	Emergent	5	1	?	NA, DP	-	Dead	-	-	?	AVR (21)	114 / 65	76 / 44
E3	77	F	Hypertension	-	?	Emergent	0	3	?	NA	Yes	Alive	?	?	-	-	124 / 95	89 / 55
E4	83	M	AF, HF, Hypothyroidism	TR + MR + AR +	Preserved	Emergent	5	10	23	NA	Yes	Alive	?	?	-	TAVI	73* / 29	49* / 23
E5	81	M	CKD, Hypertension, Type 2 DM, Previous stroke	-	Severe depression	Emergent	26	10	12	-	-	Alive	?	?	-	-	41 / 26	26 / 15
E6	75	M	Hypertension, HF, Previous stroke	-	Mild depression	Emergent	0	26	5	NA	Yes	Alive	Alive	?	-	TAVI	59 / 35	44 / 23
E7	74	M	Hypertension, Type 2 DM, AF, HF	-	Preserved	Emergent	9	1	12	-	-	Dead	-	-	Cardiogenic shock	-	46 / 26	37 / 21
E8	91	M	Hypertension, HF, Previous AMI	TR + MR ++ AR +	Mild depression	Emergent	0	4	11	NA	Yes	Alive	Alive	Alive	-	TAVI	49 / 31	38 / 26
E9	66	M	HF	TR ++ MR ++ AR +	Severe depression	Emergent	2	1	0	NA	Yes	Dead	-	-	Cardiogenic shock	-	?	?

Patient characteristics

In emergent cases, one-third were female while in urgent cases, female patients comprised 60%. The mean age was 77.11±7.64 years on emergent cases and 74.6±6,9 years on urgent cases (p=0.55). There was a significant percentage of patients referred from other hospitals, 44% of emergent and 40% of urgent cases.

Regarding patients’ co-morbidities, baseline depression of the left ventricle ejection fraction was present in 88.8% of emergent patients and in all urgent patients, being severe in 22.2% of emergent cases and in 60% of urgent cases. Only one patient, deemed as urgent, had been previously submitted to BAV. The procedure took place four years before, following a surgical refusal, and he experienced significant aortic valve restenosis meanwhile.

Cardiogenic shock characteristics and decision to intervene

Emergent cases had a mean time between diagnosis and cardiac intensive care unit admission of 0.1±2.9 days while urgent cases were admitted in 9.4±8.0 days (p=0.05). Emergent cases were submitted to BAV within 1.1±1.2 days while urgent cases took 16.2±9.7 days from diagnosis to intervention (p=0.025).

The Euroscore II of emergent cases was higher than that of urgent cases, 19±7% vs 11±5% (p=0.037), respectively.

**Figure 1:**
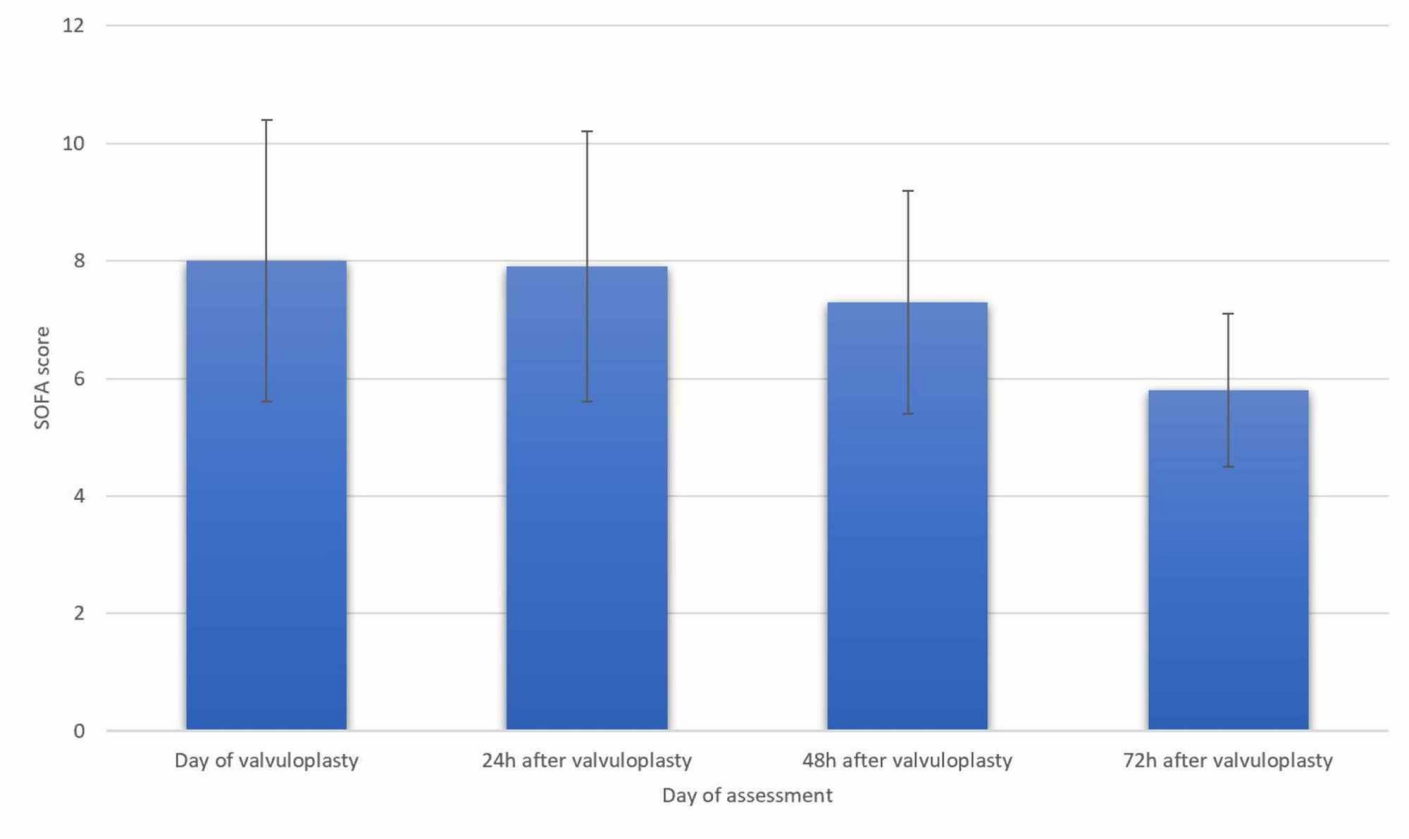
SOFA score evolution in emergent cases (n=9) SOFA - sequential organ failure assessment

The SOFA score on the day of BAV was also higher in emergent cases, 8.0±2.4, than in urgent cases, 4.8±2.9 (p=0.047). A decrease in SOFA score was seen on the days after the procedure in emergent cases, from 8.0 to 5.8, although not statistically significant (p=0.27), as seen in Figure 1.

On the day of BAV, six emergent cases (67%) and one urgent case (20%) were on vasopressors, mostly noradrenaline.

In emergent cases, the mean number of days of invasive mechanical ventilation was 0.56±0.73 days before the procedure and 2.44±3.24 days after the procedure. Urgent cases had an even lower period of invasive mechanical ventilation. On the day of the procedure, 44.4% of emergent cases were on invasive mechanical ventilation versus none in urgent cases.

**Figure 2:**
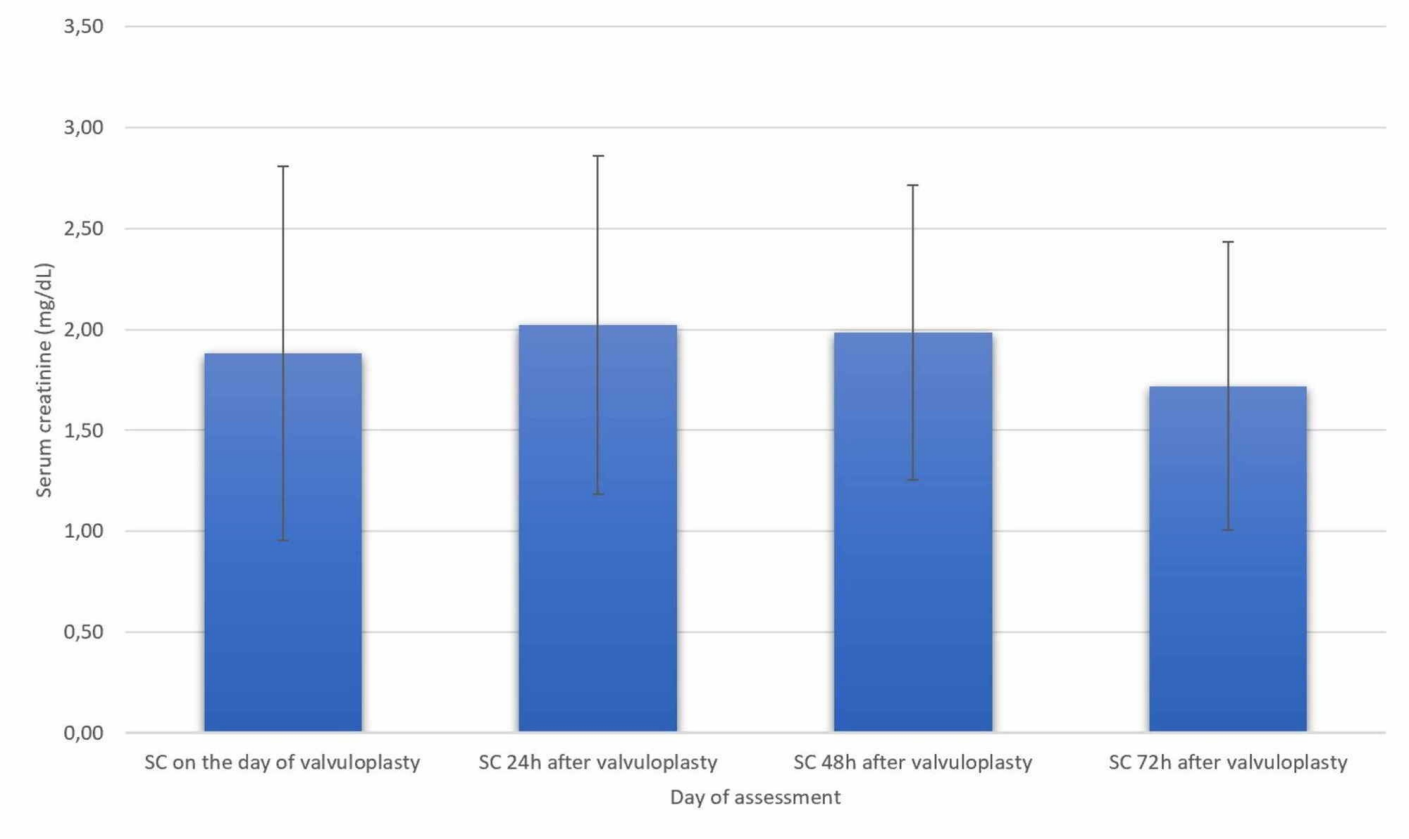
Serum creatinine variation in emergent cases (n=9) SC - serum creatinine

Before the procedure, the mean creatinine was 1.88 mg/dL in emergent cases and 1.19 mg/dL in urgent cases while it was 1.72 mg/dL and 1.31 mg/dL, respectively, 72h after the procedure. A decrease was noted for emergent cases, although not statistically significant (p=0.58), as seen in Figure 2.

Procedural data

All procedures used Loma Vista’s TRUE Dilation balloon catheter (Burlingame, CA, US). The arterial puncture site was closed with either the Perclose Proglide® suture-mediated closure system (Abbott Laboratories, IL, US). or manual and mechanical compression. There was no noteworthy aortic regurgitation (that is, more than mild post-procedure aortic regurgitation) or arterial puncture site vascular complications after BAV, whether emergent or urgent.

The average peak and mean trans-aortic gradients before the procedure were 73.13±31.27 mmHg and 51±21 mmHg for emergent cases while they were 43±14.78 mmHg and 31.75±15.59 mmHg for urgent cases (p=0.07 and 0.14, respectively). The mean reduction of the peak trans-aortic gradient was 42±12% for emergent cases and 56±16% for urgent cases (p=0.10).

Outcomes

The mean number of days in the CICU was 7.2±8.0 days for emergent cases and 5.8±4.4 days for urgent cases (p=0.72). The median number of days in the hospital after discharge from the CICU was 11 (interquartile range (IQR)=7) days for emergent cases and 15.5 (IQR=9) days for urgent cases.

No patients were readmitted for restenosis, although most procedures were done quite recently, so some procedures lack enough follow-up period. Peri-procedure mortality, within a day after BAV, was 11.1% for emergent cases (one patient) and 0% for urgent cases.

The 30-day mortality rate was 33% for emergent cases and 0% for urgent cases (p=0.14). In emergent cases, one-year mortality was 33%, whereas for urgent cases, one-year mortality was 80% (p=0.19). Even among patients who died during follow-up, mostly following refractory cardiogenic shock, one patient in the emergent group survived long enough to have her aortic valve surgically replaced, and another patient in the urgent group survived long enough to be submitted to TAVI.

Bridge to TAVI or AVR

Of all patients, six were deemed candidates for TAVI or AVR. In emergent cases, four patients were later submitted to TAVI while one patient had AVR. In urgent cases, one patient was referenced to TAVI.

## Discussion

The results show that for all BAV performed in the last 11 years in our institution, only in the last four years was this procedure done to patients in cardiogenic shock with severe AS, just three years after the European Society of Cardiology’s 2012 guidelines started recommending it [17].

There are at least eight studies published on this topic since Cribier published his first results with 10 patients in 1992 [22]. Except for Debry and colleagues’ work, which is the latest study, all previous papers present no more than two dozen patients in single-center studies, while using different definitions for cardiogenic shock and employing logistic Euroscore for patient characterization, instead of the more recent Euroscore II. Olasinska-Wisniewska and colleagues also report cases of patients in cardiogenic shock using the latest Euroscore II, although they make no distinction between patients with cardiogenic shock and pulmonary edema due to severe heart failure [23]. Some report the peak aortic gradient change while others report the mean aortic gradient change after BAV. Hence, there is considerable methodological variability in the already few reports available.

As our study was retrospective, we made an effort to include as many details as possible, which previous studies failed to include consistently. Yet, we were unable to do so due to the high number of patients referenced from other hospitals for BAV, which made it difficult to acquire data before the procedure as well as follow-up data.

Unlike previous studies, we chose to separate cases into urgent and emergent, as this is more clinically relevant, as shown by the difference in patient characteristics, and then measured their outcomes separately. The results obtained from this division may prove useful for centers who have no immediate access to BAV. Urgent patients, as emergent patients, presented or developed cardiogenic shock during their hospital stay, yet they were treated medically until BAV was scheduled, as their clinical status was improving with medical therapy alone but they were unfit for TAVI or their status worsened after sustained improvement with medical therapy.

Compared with the only study that characterized patients with Euroscore II by Debry and colleagues [22], patients in this study had significantly less operative risk. Comparing our study’s patient characteristics with the former study, there are fewer patients with chronic obstructive pulmonary disease, less renal impairment, and less extracardiac arteriopathy. However, the SOFA score of emergent patients on the day of BAV in our study was higher than the ones from Debry’s study (8.0±2.4 versus 6.0±2.9). This may mean that our study’s patients deemed emergent were clinically worse upon BAV, yet had fewer baseline comorbidities. There were more emergent patients in our study that were already on invasive mechanical ventilation upon BAV (44% versus 13%), for example. Also, the mean time to BAV in our emergent patients was 0.1±2.9 days, less than in the hypotensive or non-hypotensive patients in Debry’s study.

As for aortic valve gradients, the mean reduction was within the values regarded as for a successful procedure [11]. Our emergent patients' mean aortic gradient before the procedure was similar to Cribier’s patients [7] and higher than Debry’s [22]. More than half of emergent patients had values higher than 60 mmHg, a cut-off value proven to be associated with even greater mortality [24].

Previous reports state that BAV performed until 48h of onset of cardiogenic shock or start of inotropic agents is associated with better outcomes [5,22]. Our emergent patients belong in this group, and their 30-day mortality was 33%, less than that reported in those reports, although one of them dates back to 2001 [5]. Thirty-day mortality seems to be decreasing over the years [22] although there is some variability in recent studies. More recently, Hamid and colleagues report that of their four patients in cardiogenic shock with severe AS, none survived the periprocedural period, yet they present less information on these patients [25].

Our urgent patients, who presented with cardiogenic shock just as the emergent ones, yet were medically treated first, had less organ dysfunction on the day of BAV, and all survived the first 30 days after the procedure. However, only one patient needed a vasopressor and none was given inotropic medication during their stay in the hospital. The patient that needed a vasopressor had previously been admitted to another hospital with acute pulmonary edema, having had vasopressor support and invasive mechanical ventilation, both of which were removed successfully until the patient decompensated again. This is also compatible with previous reports [5,22], and this patient suggests that medically, therapy alone may not be enough in the short term.

It is noteworthy, nonetheless, that 80% of urgent patients were deceased just one year after BAV and none seem to have died from cardiogenic shock to the best of our knowledge. Five out of nine patients initially submitted to emergent BAV had later definitive correction of aortic stenosis, either by TAVI or AVR. The remaining three patients were deemed unfit for TAVI due to their comorbidities. Hence, patient selection, based on their previous medical condition, allowed for their survival past the acute cardiac phase, without affecting their mortality from cardiac causes.

Although previously stated that TAVI has technical limitations that may preclude its use in emergent settings, Frerker and colleagues recently accessed the outcomes of emergent TAVI (eTAVI) in 27 patients with cardiogenic shock [26]. Not only was the procedure possible in hemodynamically unstable patients but also the 30-day mortality rate was 33,3%, very much similar to our study. A comparison of patients’ severity of shock is difficult, however, as the authors did not present their SOFA scores, although they did include patients on mechanical ventilation and used the same definition of cardiogenic shock as we did. The authors also relied on a logistic Euroscore for risk stratification. To achieve eTAVI, the authors did not perform a CT scan, choosing to take measurements of the aortic valve annulus using transesophageal echocardiography and angiography. Cardiopulmonary bypass was necessary in 25.9% of patients, a procedure that none of our patients required and neither did Debry’s patients. Another recent report showed successful TAVI in cardiogenic shock, although the patient was put on ECMO prior to the procedure, again underscoring that extracorporeal support may be necessary for a successful procedure [3]. Bongiovanni and colleagues compared eTAVI with emergent BAV, having shown no significant difference in mortality, although they cannot rule out selection bias, which could have made eTAVI mortality lower. They did note significantly more vascular complications and stroke after eTAVI than emergent BAV [15]. On one hand, our approach does imply submitting patients to two separate procedures, but on the other hand, a smaller number of potentially futile definitive treatments may be performed.

As for AVR, three cases been reported of successful surgical aortic valve replacement in patients presenting in cardiogenic shock with severe AS, all alive after six months [16].

Therefore, unlike previously stated in the literature, all three procedures seem feasible in cardiogenic shock patients. BAV allows for patients in shock with multiple organ dysfunction to survive to TAVI or AVR. eTAVI, although technically possible, is associated with the need for a cardiopulmonary bypass in a quarter of cases [26]. Emergent AVR has already proven itself safe in three cases. What is now needed is to understand in which cardiogenic shock situations each procedure has the most benefits, for which a comparison study is needed, as studies to date are methodologically different.

Our study has several limitations, some of which have already been mentioned. It is a retrospective study, for which limited information was available and not all parameters could be obtained. Namely, SOFA and Euroscore II were calculated by the authors from information in each patient’s file. The fact that a proportion of patients were referenced from other hospitals and then sent back after a few days in our CICU made obtaining follow-up information difficult. The low number of patients for each group only allowed us to be descriptive relative to the findings, although they are in concordance with previous studies. Also, although we choose to make the division between emergent and urgent, we found no written justification for postponing BAV on patients’ medical records, although the medical reasons could be understood from the description of the patients’ status.

## Conclusions

BAV in select patients with cardiogenic shock and severe aortic stenosis provides a valuable bridge to the decision, being a readily available procedure with an acceptable benefit/risk profile in such adverse clinical conditions. The peri-procedural and short-term mortality are largely related to ominous clinical presentation, frequently with profound shock and multiorgan failure, and the technical aspects of the procedure seem to have been limited to no impact on the so-called hard outcomes. There may be an important role in the earlier performance of BAV in this subset of patients, as it may promptly improve cardiocirculatory, respiratory, and renal failure and preclude the entrance in an irreversible stage of cardiogenic shock. This “time-to-BAV” concept might be the critical factor to improve clinical outcomes with the technique when facing the increasingly complex and critically ill cardiovascular patients.

eTAVI is now technically feasible, although it seems associated with the need for a cardiopulmonary bypass. This aspect may tilt the table towards BAV in select emergent cases, but further studies are needed, given the heterogeneity of this challenging population and hence of the available literature.
